# The Examination Fetish

**Published:** 1907-03-16

**Authors:** 


### The Hospital

A JOURNAL OF

The Medical Sciences and Hospital Administration.

VOL. XLI.—No. 1070. SATURDAY, MARCH 16, 1907.

### THE EXAMINATION FETISH.

THE life and work of John Hunter have formed texts for many orations, and from them lessons and morals almost innumerable have been drawn. In a recent address Dr. Lauriston E. Shaw has added to this long list, and has ingeniously made one or two incidents in Hunter's career the basis for a critical attack on the existing methods of medical education and examination. The defective school training of the great surgeon and anatomist suggests to Dr. Shaw that an ordinary scholastic and college discipline might have "changed one of our most brilliant thinkers into a merely successful practitioner." And Hunter's energetic protest, at the very close of his life, against the exclusion from his teaching of those whose preliminary education had not reached the level of the official standard, is advanced as a demonstration that he himself recognised such a policy as both arbitrary and unsound. From these allusions it may possibly be anticipated that Dr. Shaw's ambition is to sweep away all compulsory examinations in general knowledge as necessary preliminaries to entrance on the medical curriculum. But here even his reforming hand—and it is one which, as will appear immediately, does not want in vigour—is stayed, presumably by the logic of facts. He desires, on the contrary, that the preliminary education of the medical student shall be as broad and general and non-technical as possible. Yet it may fairly be said that if the incidents quoted from Hunter's career afford a valid basis for the organisation of modern medical education, they teach that the demand for a preliminary examination, or, for the matter of that, for instruction in general knowledge, is unnecessary or even harmful. Dr. Shaw apparently shrinks from this conclusion, and thus, so far as the force of Hunter's example is concerned, his argument hardly manages to stand upright. The fact is that Hunter was a genius, who would doubtless have made his power effective under almost any conditions. What he did, or did not do, is not a safe basis on which to rest regulations for the government or encouragement of average minds.

Dr. Shaw's main thesis, however, is independent of the illustrations which he draws from Hunter's career. It is, in brief, that the existing examination system exercises a blighting influence upon both teachers and pupils throughout the whole course of the medical curriculum. The teacher is driven, in spite of himself, to neglect sound methods for the purpose of cramming students for their examinations. And the student is compelled to accumulate in his memory facts which "pay," rather than to devote time to the cultivation of his powers of observation and judgment—it is descriptions of things in books, rather than the things themselves, which he is forced to compass. When all this is said by a teacher of twenty years' experience on the staff of one of the leading metropolitan medical schools, it may well merit attention. And in substance, if not in detail, its accuracy will hardly be questioned by those familiar with the facts. The suggested conclusion, Dr. Shaw does not hesitate to affirm, is, that examinations as they at present exist must be abolished. The alternative suggested is that records of each student's practical work at the bedside, in the post-mortem room, and in the laboratory, should be made and preserved, and that these should be subject to surprise visits from an official inspector whose duty it shall be to see that the work is both properly done and fairly marked. There will be few earnest clinical teachers who would object to an arrangement which would place proper emphasis on the necessity for practical work performed by the student himself, and such an arrangement is quite consistent with the present examination system. It might be advocated and pressed as good and necessary in itself, and need not be associated with a crusade against examinations in general.

We will venture to suggest that a proposal to abolish examinations is not a practical policy. That, like other human contrivances, they are fallible and have defects, cannot be denied. But they have cured certain evils, they exist, and they are capable of improvement. Dr. Shaw recognises the principle that a teacher ought to take some part in the examination of his own pupils, granted that an examination of any kind is advisable. He regrets that the exclusion of the teacher is much the commoner plan, a statement which does not apply to the Scottish and provincial universities. He regrets also that examinations are so largely concerned with verbal descriptions, rather than with practical tests. In both of these positions he is sure of abundant sympathy and support. But to attempt to abolish the entire examination system is, we believe, a vain and hopeless undertaking. Reforms in the directions Dr. Shaw indicates are not only advisable but possible. Persistence will, we think, secure that the claim of the teachers in the London schools shall be recognised as they are recognised elsewhere. And regulations which emphasise the value of practical tests can be defined and enforced by the necessary authorities. Modifications of examinations on these lines, together with the inspection of the student's work during the curriculum, would, we doubt not, produce admirable results. But, like many other good things, these proposals will cost money. And even at present, teachers and examiners receive remuneration on anything but a liberal scale.

